# Genomics and Metagenomics in Microbial Ecology: Recent Advances and Challenges

**DOI:** 10.1264/jsme2.ME3201rh

**Published:** 2017-03

**Authors:** Takashi Narihiro, Yoichi Kamagata

**Affiliations:** 1 Bioproduction Research Institute, National Institute of Advanced Industrial Science and Technology (AIST)Tsukuba Central 6, Tsukuba, Ibaraki 305–8566Japan

In the book “*What is Life?*,” physicist Erwin Schrödinger (24) expected that:

“To give the statement life and colour, let me anticipate what will be explained in much more detail later, namely, that the most essential part of a living cell-the chromosome fibre may suitably be called an aperiodic crystal.”

Ten years later, Watson and Crick discovered that the “aperiodic crystal,” named deoxyribonucleic acid (DNA), had a double helix structure consisting of nucleotides (adenine, thymine, cytosine, and guanine) and a sugar-phosphate backbone (34). A gene is a sequence of DNA that encodes functional molecules (*e.g.*, RNAs and proteins). A genome is the entire set of genes of an organism and a blueprint of physiological and biochemical functions that are expressed by an organism. To date, the genes and genomes of diverse living organisms have been sequenced by a large number of research teams. As of February 2017, more than 100,000 genomic sequences including those of 89,843 prokaryotes, 3,976 eukaryotes, and 7,088 viruses, have been deposited in the NCBI GenBank database (Fig. 1) (1). The widespread use of high-throughput sequencing technologies has increased the number of genomes of prokaryotes that have been sequenced in the last two years (>25,000 genomes per year). In addition, more than 1,000 metagenomic studies are available through the JGI Integrated Microbial Genomes & Microbiomes (IMG/M) web server (Fig. 2) (9). These metagenomic sequences are prompting researchers to attain a clearer understanding of the ecophysiology of yet-to-be cultivated organisms in environments (*e.g.*, marine, freshwater, and soil), engineered systems (*e.g*., waste/wastewater treatment process), and host-associated microbiomes (*e.g.*, humans, mammals, and Arthropoda) (4, 12).

Some of the most recent outcomes are (meta)genome-based insights into syntrophy in anaerobic methanogenic ecosystems. Syntrophic substrate metabolizers (syntrophs) play an important role in the oxidation of by-products (*e.g.*, volatile fatty acids, primary alcohols, and amino acids) from the fermentative degradation of high-molecular-weight organic compounds in methanogenic ecosystems (6, 13, 14, 23). Extensive efforts have been made to elucidate the mechanisms underlying syntrophic degradation pathways and energy conservation systems based on the genomes of the cultured representatives of syntrophs (26). Nobu *et al.* (17, 19) constructed the draft genome of *Syntrophorhabdus aromaticivorans* strain UI, isolated from a methanogenic isophthalate-degrading enrichment culture (22), and identified syntrophic degradation pathways for benzoate, phenol, and terephthalate. This achievement was the first explicit genome-based analysis of anaerobic phenol and terephthalate metabolism, which had previously remained unclear. The strain UI genome encodes flavin oxidoreductase-heterodisulfide reductase (Flox-Hdr), electron transfer flavoproteins (ETFs), hydrogenases, and formate dehydrogenases, which appear to be closely associated with the generation of H_2_ and formate, important intermediates for the interspecies electron transfer underpinning anaerobic syntrophy. More recently, comparative genomics of three syntrophic bacteria: “*Syntrophomonas wolfei* subsp. *methylbutyratica*” strain 4J5 (15), *Syntrophothermus lipocalidus* strain TGB-C1 (2), and *Syntrophomonas wolfei* subsp. *wolfei* strain Göttingen (25) elucidated the metabolic pathways of branched-chain fatty acids (BCFAs) such as 2-methylbutyrate and isobutyrate (16). Anaerobic BCFA metabolism remained unclear even though BCFAs are important products from amino acids. These findings indicate that the genomes of BCFA-degrading strains 4J5 and TGB-C1 encode unique β-oxidation-related enzymes for syntrophic BCFA oxidation as well as various energy conservation systems including ETF-linked acyl-CoA dehydrogenase, ETF-linked iron-sulfur (Fe-S) binding reductase, ETF dehydrogenase (FixABCX), and Flox-Hdr. Manzoor *et al.* (7) constructed the draft genome of the acetate-oxidizing syntroph *Syntrophaceticus schinkii* strain Sp3. The whole transcriptome profiling of co-cultures of strain Sp3 and *Methanoculleus bourgensis* fed with acetate revealed that genes involved in the Wood-Ljungdahl pathway, hydrogenases, ATP synthase, formate dehydrogenases, and acetate transporters, were clearly expressed, supporting its unique physiology for syntrophic acetate oxidation (8). A metagenomic approach may lead to the identification of methanogenic microbiota that contain, to a large extent, fastidious syntrophs that have never been cultivated. Wawrik *et al.* (35) performed metagenomic sequencing of an uncultured *Smithella*-predominated methanogenic enrichment culture capable of mineralizing long-chain *n*-paraffins (C28–C50). They demonstrated for the first time that the uncultured *Smithella* sp. SDB metagenome encoded the alkylsuccinate synthase gene (*assA*) for the degradation of *n*-paraffins, while ETF-linked Fe-S oxidoreductase, periplasmic formate dehydrogenase, and hydrogenase were used for energy conservation. By using metagenomics together with metatranscriptomics and single-cell genomics, Nobu *et al.* (18) demonstrated that phylogenetically diverse microbes cooperate in the synergistic degradation of terephthalate, catabolic by-products (acetate, butyrate, and H_2_), and detritus compounds (*e.g.*, amino acids and glycerol) in a methano-genic bioreactor; *i.e.*, *Pelotomaculum* and *Syntrophorhabdus* degrade terephthalate to acetate; *Thermotogae* members syntrophically oxidize acetate; *Syntrophaceae*, “*Ca.* Marinimicrobia” (SAR406), and “*Ca.* Hydrogenedentes” (NKB19) may play roles in the degradation of butyrate/BCFAs, amino acids, and glycerol, respectively; “*Ca.* Atribacteria” (OP9) and “*Ca.* Cloacimonetes” (WWE1) are identified as syntrophic propionate oxidizers. This study successfully provided a comprehensive description of a complex syntrophy-associated microbiota in order to more clearly understand the metabolic capacities of as-yet uncultivated organisms, named “microbial dark matter.”

A metagenomic approach is frequently used to analyze host-microbe interactions in mammals, plants, and other living organisms (Fig. 2) (3). Minami *et al.* (10) conducted a comparative analysis of 29 genomes of *Methylobacterium* spp. belonging to three 16S rRNA gene-based phylogeny groups (Groups I, II, and III) derived from soybean stem samples, and found that Group I *Methylobacterium* members were dominant in soybean-associated *Methylobacterium* and possessed a complete set of genes for the degradation of ureide compounds (allantoin and allantoinate), implying competition between *Methylobacterium* spp. and soybean plants in the utilization of ureide compounds as the nitrogen source. In contrast, the genomes of two strains associated with Group II *Methylobacterium* lacked the genes of ureidoglycorate urealyase, the last step of ureide degradation. This genome-based evidence suggests that the survival strategy of *Methylobacterium* spp. in the rhizosphere and phyllosphere is metabolically diverse. Metagenomic analyses of the bacterial community of the taproot of sugar beet revealed that the genes involved in plant growth-promoting traits (PGPTs) (*i.e.*, plant disease suppression, phosphate solubilization, methanol utilization, and 1-aminocyclopropane-1-carboxylic acid [ACC] deaminase) were frequently assigned to the genera *Mesorhizobium*, *Bradyrhizobium*, and *Streptomyces*, suggesting their important roles in the taproot of sugar beet (31).

Viral genome data has been accumulated due to the importance in clinical and biological aspects (Fig. 1) (5, 21, 27). Takemura (28, 29) discovered a novel DNA virus, named *Tokyovirus*, of the family *Marseilleviridae* from water/soil samples collected from Arakawa River in Tokyo. A total of 487 coding sequences (CDSs) were identified from the *Tokyovirus* genome, and most CDSs exhibited high homology with other *Marseilleviridae* family members, while several putative CDSs were found to be functionally unknown *Tokyovirus*-specific CDSs, implying that *Tokyovirus* evolved separately from the previously identified members of the *Marseilleviridae* family. Oh *et al.* (20) reconstructed the draft genomes of virophages of inland lakes from metagenomic sequences and found a novel virophage population from a planktonic microbial community of the Tibetan mountain lake, Lake Qinghai. The Qinghai Lake virophage (QLV) genome harbors 25 open reading frames including ATPase, cysteine protease, major and minor capsid proteins, and DNA helicase/primase/polymerase, which are commonly conserved in other virophage genomes. They successfully developed a strategy to detect virophage populations using genes encoding major capsid proteins as a molecular marker. In order to facilitate studies on viral (meta) genomics, a recovery technique of viral genomes using tandem column chromatography (32) and a sequencing method for intracellular RNA viruses, so-called fragmented and loop primer ligated dsRNA sequencing (FLDS) (33), have been developed.

Morohoshi *et al.* (11) recently reported the complete genome sequence of *Pseudomonas chlororaphis* subsp. *aurantiaca* strain StFRB508, a bacterium that regulates the production of the antifungal agent phenazine through *N*-acyl-l-homoserine lactone (AHL)-mediated quorum sensing. They revealed that the StFRB508 genome encodes three quorum-sensing systems comprising a set of AHL-synthase and AHL-receptor genes (*i.e.*, *phzI*/*phzR*, *aurI*/*aurR*, and *csaI*/*csaR*), which may play an important role in the biosynthesis of phenazine. Tashiro *et al.* (30) performed genomic analyses on small colony variants (SCVs) of *Escherichia coli* with phenotypic differences such as slower growth, less biofilm formation, and greater resistance to aminoglycoside antibiotics than the parent strain. The findings obtained indicated that the transposon Tn*1000*, which was originally located in the conjugative F plasmid, was inserted into the *hemB* gene locus in the chromosome, suggesting that SCVs are caused by inactivation of the heme biosynthetic pathway. These findings strongly suggest that the F plasmid has a role in the appearance of genetic variants as a result of DNA recombination within a single cell.

In “*What is Life?*,” Schrödinger discussed the order of the mechanisms of life as follows (24):

“The orderliness encountered in the unfolding of life springs from a different source. It appears that there are two different ‘mechanisms’ by which orderly events can be produced: the ‘statistical mechanism’ which produces order from disorder and the new one, producing order from order.”

In recent years, vast amounts of genomic and metagenomic sequencing data have been generated and applied to elucidate the novel ecophysiological functions of living organisms; however, researchers are inclined to ignore sequences that have neither conserved regions, motifs, nor known arrangements, and consider them to be disordered or disorganized. Since a large amount of omics-derived data is accumulating, finding order from disorder will represent the next challenge for researchers.

## Figures and Tables

**Fig. 1:**
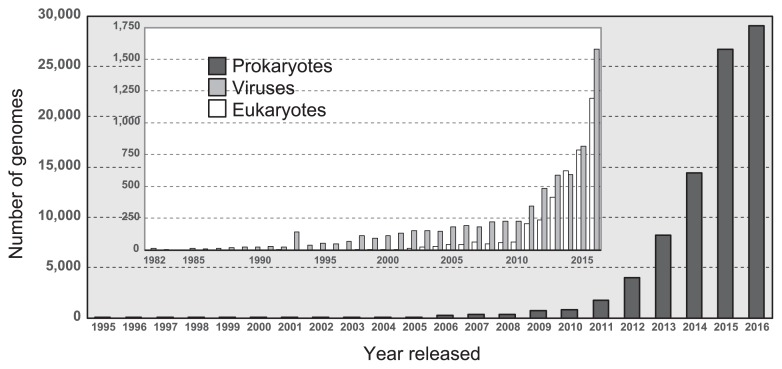
Number of genomes of prokaryotes, eukaryotes, and viruses sequenced each year based on the NCBI GenBank database (1).

**Fig. 2:**
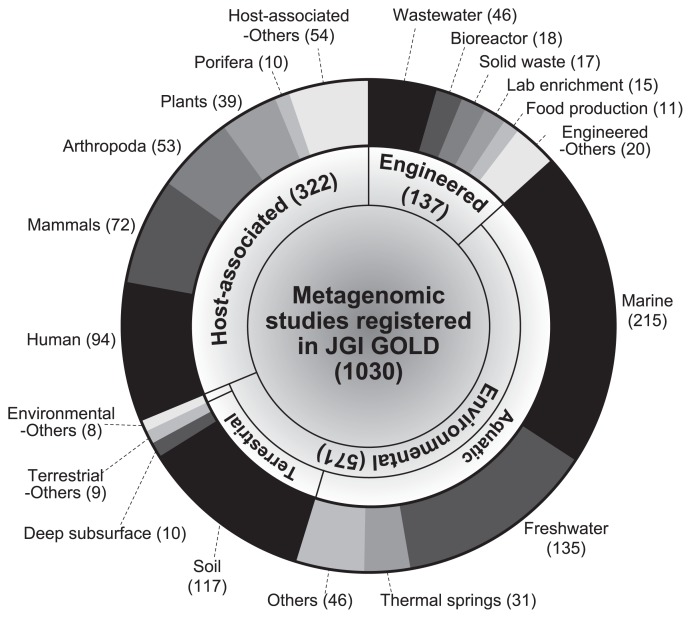
Number of metagenomic studies in the JGI Integrated Microbial Genomes & Microbiomes (IMG/M) web server (9) as of February 2017. Category and subcategory names are according to the IMG/M database.
